# Effects of Space Dimensionality within Scaffold for Bone Regeneration with Large and Oriented Blood Vessels

**DOI:** 10.3390/ma16247518

**Published:** 2023-12-05

**Authors:** Koichiro Hayashi, Ryo Kishida, Akira Tsuchiya, Kunio Ishikawa

**Affiliations:** Department of Biomaterials, Faculty of Dental Science, Kyushu University, 3-1-1 Maidashi, Higashi-ku, Fukuoka 812-8582, Japan; kishida@dent.kyushu-u.ac.jp (R.K.); tsuchiya@dent.kyushu-u.ac.jp (A.T.); ishikawa@dent.kyushu-u.ac.jp (K.I.)

**Keywords:** scaffold, dimension, apatite, 3D printing, bone, orientation

## Abstract

The internal structure of the scaffolds is a key factor for bone regeneration. In this study, we focused on the space dimensionality within the scaffold that may control cell migration and evaluated the effects on the size and orientation of blood vessels and the amount of bone formation in the scaffold. The carbonate apatite scaffolds with intrascaffold space allowing one-dimensional (1D), two-dimensional (2D), or three-dimensional (3D) cell migration were fabricated by 3D printing. These scaffolds had the same space size, i.e., distances between the struts (~300 µm). The scaffolds were implanted into the medial condyle of rabbit femurs for four weeks. Both the size and orientation degree of the blood vessels formed in the scaffolds allowing 1D cell migration were 2.5- to 4.0-fold greater than those of the blood vessels formed in the scaffolds allowing 2D and 3D cell migration. Furthermore, the amount of bone formed in the scaffolds allowing 1D cell migration was 1.4-fold larger than that formed in the scaffolds allowing 2D and 3D cell migration. These are probably because the 1D space limited the direction of cell migration and prevented the branching of blood vessels, whereas 2D and 3D spaces provided the opportunity for random cell migration and blood vessel branching. Thus, scaffolds with 1D space are advantageous for inducing large and oriented blood vessels, resulting in a larger amount of bone formation.

## 1. Introduction

The aging of the population worldwide has led to an increase in surgery for knee osteoarthritis, that is high tibial osteotomy [1,2,3], and bone fractures caused by falls and traffic accidents. Therefore, there has been an increased demand for materials and cellular scaffolds to regenerate bones faster [4,5]. Various scaffolds have been developed for use in bone regeneration, and the typical composition of the scaffold is calcium phosphate [5,6,7]. Carbonate apatite, an analog of bone mineral, has been reported to achieve earlier bone regeneration than other calcium phosphates, such as hydroxyapatite (HAp) and beta-tricalcium phosphate (β-TCP) [8,9]. In addition, most scaffolds contain spaces, i.e., pores or channels, that facilitate cell penetration [10,11,12,13].

Conventionally, porous scaffolds are fabricated by porogen leaching [14,15,16,17], gas formation [18], negative replication of the template [19,20,21,22,23], and connection of granules [24,25]. However, these methods have low reproducibility, and it is difficult to control the pore characteristics that are crucial for promoting cell penetration into the scaffold. Recently, advances in three-dimensional (3D) printing technology have allowed direct control of the scaffold structure and increased the degrees of freedom in the design [26,27,28,29,30,31,32,33]. Therefore, scaffolds with various pore characteristics such as structure, size, and porosity can be fabricated.

Various types of scaffolds with 3D porous structures, such as lattice and grid structures, have been fabricated using 3D printing technology [33,34,35,36,37,38,39,40,41]. Compared to scaffolds with one-dimensional (1D) porous structures such as the honeycomb structure, scaffolds with 3D porous structures have been considered more favorable because the spaces in the 3D porous structure are open on all surfaces, whereas the spaces in the 1D porous structure are open on two specific surfaces [42,43,44,45,46,47,48]. However, it is conceivable that a 3D porous structure allows the random migration of cells and branching of blood vessels in all directions within the scaffold, which may decrease the vessel size and orientation. Although the bone formation in scaffolds with two-dimensional (2D) porous structures is unclear, in principle, these scaffolds can also provide opportunities for the random migration of cells and branching of blood vessels. In contrast, in a 1D porous structure, the directions in which blood vessels can run are limited, and this may have a positive effect, preventing the branching of vessels and leading to the formation of large and oriented vessels. Reportedly, honeycomb scaffolds can form large and oriented blood vessels, which supports the above hypothesis [42,44,47,48].

In this study, I will start a study to test the hypothesis. To validate the above hypotheses on the dimensionality of intrascaffold space, carbonate apatite scaffolds with three different types of intrascaffold spaces, that is, scaffolds with space allowing 1D, 2D, and 3D migration of cells (1D-, 2D-, and 3D-scaffolds, respectively), were fabricated via 3D printing. Through in vivo experiments using these scaffolds, the effects of the dimensionalities of the intrascaffold space on the size and orientation of blood vessels and bone formation were evaluated.

## 2. Materials and Methods

### 2.1. Fabrication of 1D-, 2D-, and 3D-Scaffolds

The structures of the 1D-, 2D-, and 3D-scaffolds (6 mm in diameter and 3 mm in height) were fabricated based on the procedures developed in our previous report [33]. These scaffolds were designed using Fusion 360 (Autodesk, San Rafael, CA, USA). The thickness of the designed strut was 300 μm. The square aperture of the channel was 300 μm on one side. Based on this design, structures were prepared by 3D printing using a stereolithography apparatus (SLA, SZ-1100, SK Fine, Shiga, Japan). For 3D printing, a photosensitive resin (50 vol.%, SPR302, SK Fine) and calcium carbonate powder (50 vol.%, particle size: 5 μm, Sakai Chemical, Sakai, Japan) were mixed using a planetary centrifugal mixer (SK-350TV, Shashin Kagaku, Kyoto, Japan) and used as precursor. Precipitation of the calcium carbonate powder in the slurry was observed on the second day after mixing. Therefore, the slurry was used immediately after preparation to prevent precipitation. The precursor slurry was cured by the laser (wavelength: 355 nm, spot size: 15 μm, power: 7 mW) equipped with the SLA. The exposure time and scanning speed of the laser were 10 s/cm^2^ and 1000 mm/s, respectively. After the laser irradiation, structures consisting of calcium carbonate and resin were obtained. The structures were subjected to ultrasonic cleaning with ethanol for 60 s to remove uncured resin.

To remove the resin, the washed structures were heated up to 650 °C at a heating rate of 0.2 °C/min and maintained at 650 °C for 24 h, with the furnace atmosphere changed from air to carbon dioxide at 550 °C to prevent the formation of calcium oxide. Thus, calcium carbonate structures were obtained.

The calcium carbonate structures were phosphatized by immersing in a 1 mol/L Na_2_HPO_4_ aqueous solution (Fujifilm Wako, Osaka, Japan) at 80 °C for 7 d to convert the composition to carbonate apatite through a dissolution-precipitation reaction. Consequently, the 1D-, 2D-, and 3D-scaffolds consisting of carbonate apatite were obtained. The scaffolds were then washed ten times with distilled water.

### 2.2. Characterization of 1D-, 2D-, and 3D-Scaffolds

Micro-computed tomography (CT) images of the 1D-, 2D-, and 3D-scaffolds were obtained using an X-ray CT (Skyscan, Bruker Corp., Billerica, MA, USA). The microstructures of the 1D-, 2D-, and 3D-scaffolds were investigated using scanning electron microscopy (SEM, S-3400N, Hitachi High Technologies, Tokyo, Japan) at an acceleration voltage of 10 kV; the samples were sputter coated with gold-palladium using a magnetron sputtering machine (MSP-1S, Vacuum Device Co., Ibaraki, Japan). The crystal phases and functional groups of the scaffolds were identified using X-ray diffraction (XRD, D8 Advance, Bruker, Billerica, MA, USA) and Fourier-transform infrared (FTIR) spectroscopy (FT/IR-6200, JASCO, Tokyo, Japan), respectively. The carbon content in 1D-, 2D-, and 3D-scaffolds was measured by CHN analysis (CHN corder MT-6, Yanaco, Kyoto, Japan). The sample size was measured using a digital micrometer (MDH25-MB, Mitutoyo Corporation, Kanagawa, Japan).

### 2.3. Ethics Statement

Animal experiments were conducted following the procedures and ethical policies approved by the Animal Care and Use Committee of Kyushu University, Fukuoka, Japan (Approval No. A22-371-1).

### 2.4. Sample Size Calculations

Sample size calculations were performed using PS: Power and Sample Size Calculation software (version 3.1.6, released in October 2018 by William D. Dupont and Walton D. Plummer, Jr., Vanderbilt University, Nashville, TN, USA). Based on the Bonferroni method, the significance level was set at 0.0167. The planned analyses comprised a continuous response variable in independent control and experimental groups (at a 1:1 ratio). For the analyses of blood vessel size, orientation angle, and anisotropy, true differences between experimental and control means were set at 60, 50, and 0.22, respectively, and standard deviations were set at 20, 12, and 0.08, respectively. It was estimated that four samples per group were required to reject the null hypothesis (i.e., the population means of the experimental and control groups are equal) with a probability (power) of 0.8. The Type I error probability for this test of the null hypothesis was 0.017.

### 2.5. Animals

Japanese white rabbits (18-week-old, body mass: 3.0–3.5 kg) were purchased from Japan SLC (Hamamatsu, Japan). The rabbits were individually housed in cages and maintained on a standard diet with adequate water supply at the Center of Biomedical Research, Research Center for Human Disease Modeling, Graduate School of Medical Sciences, Kyushu University, Japan. Six rabbits (12 hind legs) were used in the 1D-, 2D-, and 3D-scaffold-implanted groups (4 hind legs per group).

### 2.6. Surgical Procedure

The rabbits were injected intramuscularly with ketamine (30 mg/kg) and xylazine (5.0 mg/kg), and the femoral area of both sides was shaved. The shaved femoral skin was disinfected using 10% *w*/*v* povidone-iodine (Meiji Seika, Tokyo, Japan). For local anesthesia, 2% lidocaine (0.9 mL, Showa Yakuhin Kako, Tokyo, Japan) was injected into several spots on the surgical site. The femoral condyle was exposed by making an incision (approximately 2 cm long) in the femoral skin using a scalpel. The periosteum was separated from the bone using a raspatory. Bone defects (diameter, Φ, 6 mm × depth, DP, 3 mm) were produced in the femoral epiphyses of both legs using a trephine with scale. The epiphysis lacks type H blood vessels that promote osteogenesis and angiogenesis [49,50,51], whereas the type H blood vessels are abundantly present in the metaphysis where bone defects were created in previously published studies [52,53]. Therefore, the critical size of the bone defect (Φ 5 mm × DP 10 mm) in the metaphysis should be larger than that in the epiphysis. Furthermore, the bone defect (Φ 6 mm × DP 5 mm) in the rabbit femur condyle is considered a critical-sized bone defect [54,55,56]. Even though the bone defect size (Φ 6 mm × DP 3 mm) in this study was smaller than the reported size, we have confirmed in our previous studies that the bone defect of Φ 6 mm × DP 3 mm was not spontaneously healed at 12 weeks after surgery [9,42]. In addition, the bone defect of Φ 6 mm × DP 5 mm may penetrate a portion of the epiphysis in some individuals. Therefore, the bone defect size of Φ 6 mm × DP 3 mm was selected in this study.

The 1D-, 2D-, and 3D-scaffolds were implanted into critical-sized defects. Thereafter, the incised periosteum and skin were sutured. The surgical site was disinfected with 10% *w*/*v* povidone-iodine. Finally, gentamicin sulfate solution (0.15 mL/kg, Gentacin, Takata Pharmaceutical, Saitama, Japan) was intraperitoneally injected to prevent infection.

### 2.7. Histological Analyses

Four weeks after the implantation of the 1D-, 2D-, and 3D-scaffolds, rabbit femurs (*n* = 4 per group) were collected and fixed in 10% formalin solution (Fijifilm Wako, Osaka, Japan) for 72 h. Specimens were decalcified using 0.5 mol/L ethylenediaminetetraacetic acid decalcifying solution (Fujifilm Wako, Osaka, Japan), embedded in paraffin, sectioned (3 μm in thickness), and stained with hematoxylin and eosin (HE). Histological images of the stained tissue were obtained using a microscope (BZ-X, Keyence, Osaka, Japan). The proportion of the new bone area in the bone defect and the size of blood vessels formed in the scaffold were calculated by histological analyses using the BZ-X digital analysis software (version: BZ-X800) (Keyence, Osaka, Japan). Orientation angle and anisotropy were evaluated by analyzing the stained sections using the FibrilTool, an ImageJ plug-in, according to the protocol reported by Boudaoud et al. [57].

### 2.8. Statistical Analysis

Statistical analyses were performed using EZR (Saitama Medical Center, Jichi Medical University, Saitama, Japan), a graphical user interface for R (The R Foundation for Statistical Computing, Vienna, Austria) [58]. It is a modified version of the R commander, designed to add frequently used statistical functions in biostatistics [58]. All data are presented as the mean ± standard deviation, and *p*-values < 0.05 were considered statistically significant. Multiple comparisons among all groups in the animal experiments were performed using the Tukey–Kramer test.

## 3. Results

**Figure 1:**
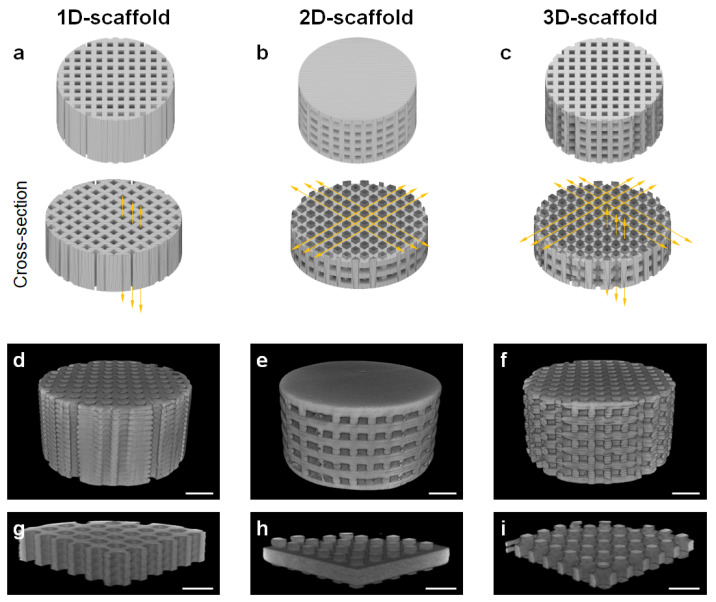
Structural designs of (**a**) 1D-, (**b**) 2D-, and (**c**) 3D-scaffolds: (top) entire bodies and (bottom) cross-sectional surfaces. Yellow arrows indicate the channel directions in which cells can migrate. Micro-CT images of the whole (**d**) 1D-, (**e**) 2D-, and (**f**) 3D-scaffolds. Micro-CT images of the cross-sectional surfaces of (**g**) 1D-, (**h**) 2D-, and (**i**) 3D-scaffolds. Scale bars: 1 mm.

To evaluate the effects of space dimensionality in the scaffold on the size and orientation of blood vessels and the new bone formation, carbonate apatite scaffolds with uniaxial channels that run along the vertical direction and can allow 1D cell migration (1D-scaffolds, Figure 1a), channels that run toward every direction along the horizontal level and can allow 2D cell migration (2D-scaffolds, Figure 1b), and combined vertical and horizontal channels allowing 3D cell migration (3D-scaffolds, Figure 1c) were designed. Micro-CT images confirmed that the 1D-, 2D-, and 3D-scaffolds were fabricated as designed (Figure 1d–f), and all channels completely penetrated the scaffolds (Figure 1g–i).

**Figure 2:**
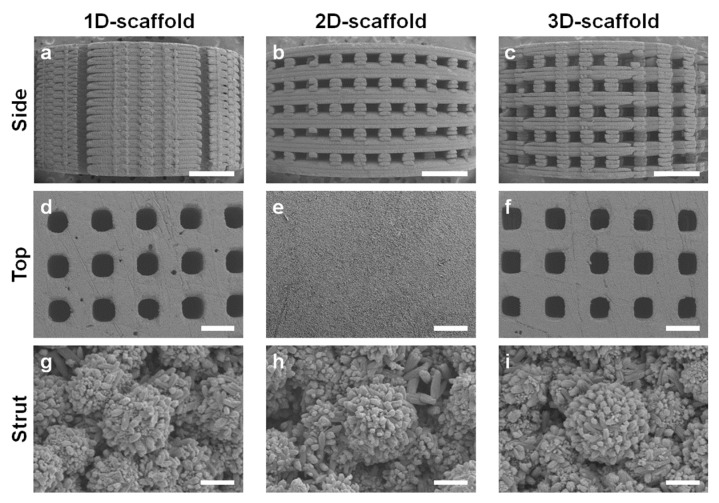
SEM images of the side surfaces of (**a**) 1D-, (**b**) 2D-, and (**c**) 3D-scaffolds. Scale bars: 1 mm. SEM images of the top surfaces of (**d**) 1D-, (**e**) 2D-, and (**f**) 3D-scaffolds. Scale bars: 500 μm. High magnification SEM images of the struts of (**g**) 1D-, (**h**) 2D-, and (**i**) 3D-scaffolds. Scale bars: 5 μm.

The SEM images show that no channels opened on the side of the 1D-scaffold (Figure 2a), whereas channels opened on the sides of the 2D- and 3D-scaffolds (Figure 2b,c). The opening sizes of the horizontal channels in the 2D- and 3D-scaffolds were 310.5 ± 8.8 and 311.6 ± 9.6 μm, respectively (Figure 2b,c). The 1D- and 3D-scaffolds possessed channel openings on the top surfaces, whereas the 2D-scaffolds did not (Figure 2d–f). The opening sizes of the vertical channels in the 1D- and 3D-scaffolds were 317.4 ± 3.9 and 315.8 ± 9.6 μm, respectively (Figure 2d,f). Thus, the opening size of the channels in the printed scaffolds was 3–6% larger than the designed channel opening size (300 μm). The strut thicknesses of the 1D-, 2D-, and 3D-scaffolds were ~340 μm, which was ~14% larger than the designed strut size (300 μm) (Figure 2a–f). The struts of the 1D-, 2D-, and 3D-scaffolds were composed of spherical aggregates consisting of rod-shaped apatite crystals (Figure 2g–i). These results demonstrate that the 1D-, 2D-, and 3D-scaffolds possessed almost equal chemical compositions and channel sizes, and only the dimensionalities of the channels differed among the scaffolds. XRD, FTIR, and CHN analyses revealed that the 1D-, 2D-, and 3D-scaffolds were composed of AB-type carbonate apatite, with 12–13% carbonate contents (Supplementary Materials, Figure S1). The compressive strengths of the 1D-, 2D-, and 3D-scaffolds were 183.6, 58.0, and 35.3 MPa, respectively.

**Figure 3:**
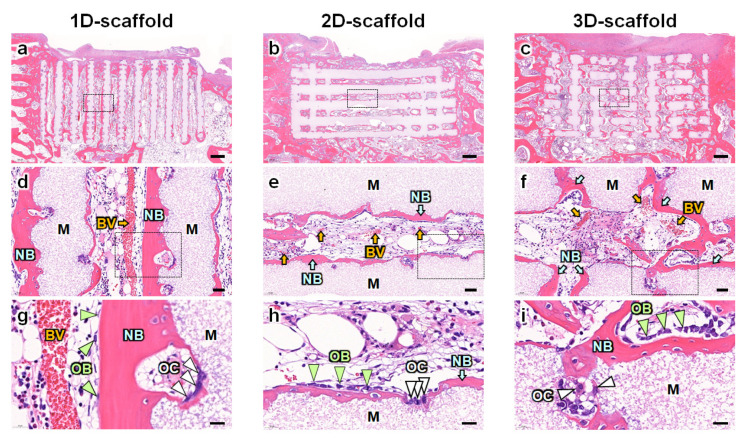
HE-stained sections of the side surfaces of (**a**) 1D-, (**b**) 2D-, and (**c**) 3D-scaffolds. Scale bars: 500 μm. High-magnification images of the regions enclosed by the squares in the images of (**d**) a, (**e**) b, and (**f**) c. Scale bars: 50 μm. High-magnification images of the regions enclosed by the squares in the images of (**g**) d, (**h**) e, and (**i**) f. Scale bars: 20 μm. M indicates scaffold material. BV and yellow arrows indicate blood vessels. NB and light blue arrows indicate new bone. OB and pale green arrows indicate osteoblasts. OC and white arrows indicate osteoclasts.

HE-stained sections showed new bone formation on the surfaces of struts in all the 1D-, 2D-, and 3D-scaffolds (Figure 3a–c). Notable differences were observed in the size and orientation of the blood vessels. In the 1D-scaffolds, large blood vessels formed in the center of the channel and were oriented uniaxially between the cancellous bone and periosteum (Figure 3d). In contrast, in the 2D- and 3D-scaffolds, the size and orientation of the blood vessels were small and random (Figure 3e,f). Furthermore, the number of blood vessels in the 2D-scaffolds was lower than that in the 3D-scaffolds (Figure 3e,f). In the 3D-scaffolds, blood vessels were mainly present at the intersection of the vertical and horizontal channels (Figure 3f). In all the 1D-, 2D-, and 3D-scaffolds, osteoblasts and osteoclasts resided on the surfaces of the new bone and scaffold struts, respectively (Figure 3g–i).

**Figure 4:**
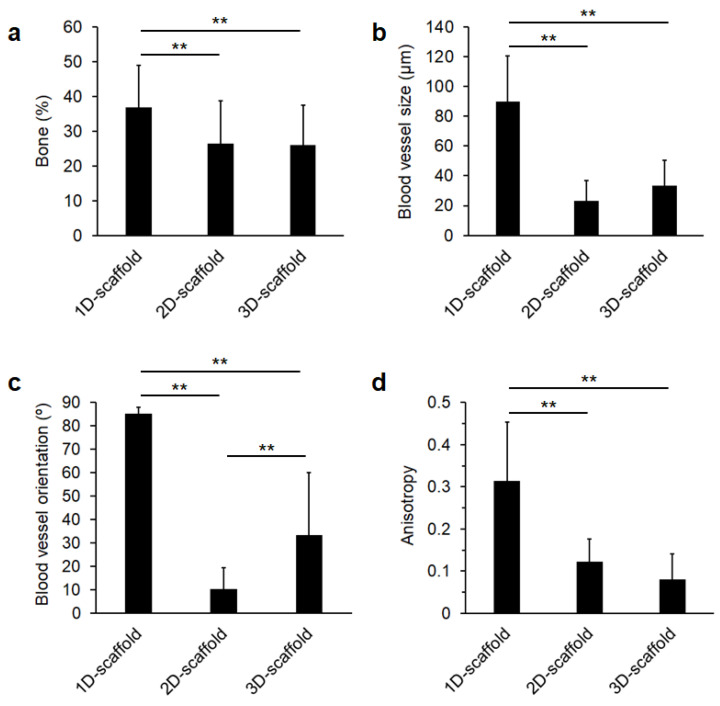
(**a**) Percentages of bone formed in the defects. (**b**) Size, (**c**) orientation angle, and (**d**) anisotropy of blood vessels formed in the scaffolds. ** *p* < 0.01.

Bone percentage in the defects in the 1D-scaffold-implanted group (36.8 ± 12.2%) was significantly higher than those in 2D-scaffold- and 3D-scaffold-implanted groups (26.4 ± 12.4% and 26.0 ± 11.4%, respectively, Figure 4a). The size of blood vessels in the 1D-scaffold-implanted group (89.7 ± 30.8 μm) was 3.9- and 2.7-fold larger than those in 2D-scaffold- (23.0 ± 13.8 μm) and 3D-scaffold-implanted groups (33.5 ± 17.2 μm), respectively (Figure 4b). The orientation of the blood vessels was defined as the angle of the blood vessels relative to the base of the scaffold. Thus, the orientations or angles were defined as 0° and 90° when the blood vessels ran horizontally and perpendicular to the base of the scaffold, respectively. The orientations of blood vessels in 1D-, 2D-, and 3D-scaffold-implanted groups were 85.2 ± 2.8°, 10.5 ± 9.0°, and 33.4 ± 26.6°, respectively (Figure 4c). Thus, the blood vessels in the 1D- and 2D-scaffolds ran parallel to the directions of their vertical and horizontal channels, respectively, and the orientation of the blood vessels in the 3D-scaffold-implanted group was intermediate between those in the 1D- and 2D-scaffold-implanted groups. Furthermore, the degree of orientation was defined as the anisotropy. The anisotropy of blood vessels in the 1D-scaffold-implanted group (0.31 ± 0.14) was 2.6- and 3.9-fold higher than those in 2D- (0.12 ± 0.05) and 3D-scaffold-implanted groups (0.08 ± 0.06), respectively (Figure 4d). Thus, the space allowing 1D cell migration produced larger and more highly oriented blood vessels than those allowing 2D and 3D cell migration. When channel intersections were present in the scaffolds, the size and orientation of blood vessels decreased.

## 4. Discussion

Ghayor et al. reported bone formation in 3D porous β-TCP scaffolds with 0.5, 1.2, and 1.7 mm pores on the rabbit calvaria [59]. The scaffolds were covered by titanium cylinders with a lid on the skin side to physically prevent the penetration of fibrous tissues into the scaffold. Therefore, in the study by Ghayor et al., fibrous tissue penetration into the scaffolds from the skin can be ignored and only bone ingrowth from the calvaria can be considered [60]. In that case, although bone augmentation did not differ by pore size, the scaffold with 1.2 mm pores showed higher osteoconduction than the scaffolds with 0.5 and 1.7 mm pores. In contrast, Guerrero et al. demonstrated that when grid-like structured TCP scaffolds with 0.4, 0.5, 0.83, and 1.25 mm pores were placed on the rabbit calvaria without covering the scaffolds by titanium cylinders, bone formation increased as pore size decreased [60]. Furthermore, we demonstrated that when carbonate apatite honeycomb scaffolds with open square channels of 230, 460, and 630 μm were placed on the rabbit calvaria without a titanium cylinder, the 230 μm channels prevented the penetration of fibrous tissues into the scaffold and promoted bone ingrowth [47]. In contrast, the 460 and 630 μm channels could not prevent fibrous tissue penetration and significantly reduced the amount of bone formation than the 230 μm channels [47]. In addition, more fibrous tissues penetrated the 630 μm pores than the 460 μm pores, resulting in lower bone formation [47]. Furthermore, channels with an opening size of approximately 300 μm were reported to be superior in ingrowths of bone and blood vessels to the channels with an opening size smaller than 200 μm, when the scaffolds were implanted into the rabbit femur epiphyses [43,46]. Thus, the scaffolds used in this study are adequate for evaluating the effects of channel dimensionalities on the size and orientation of blood vessels and bone formation.

**Figure 5:**
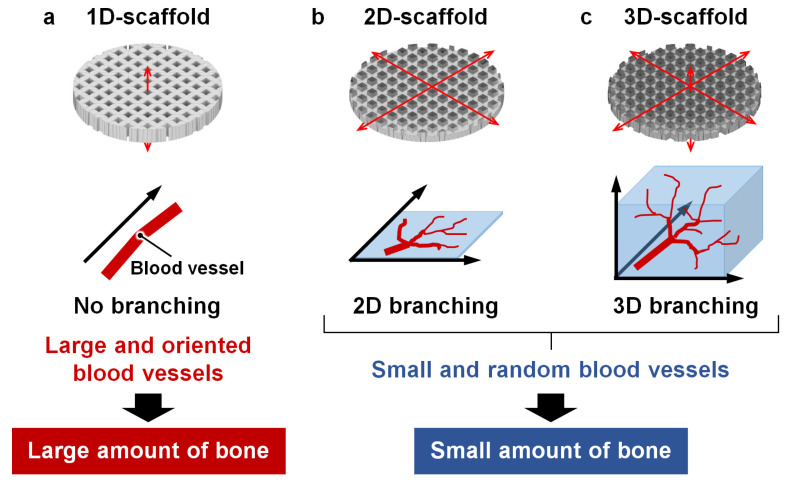
Effects of the dimensionality of intrascaffold space on characteristics of the blood vessels and bones formed in the (**a**) 1D-, (**b**) 2D-, and (**c**) 3D-scaffolds.

This study demonstrated that intrascaffold space can determine the dimensionality of cell migration, which is crucial for increasing the size and orientation degree of blood vessels formed in the scaffold. The possible reasons are as follows. In the interior of the 1D-scaffolds, the growth direction of the blood vessels is determined as the direction of the channel, thereby the branching of the blood vessels is prevented, because the space within the channel is limited (Figure 5a). Consequently, large and oriented blood vessels form in 1D-scaffolds. In the interior of the 2D- and 3D-scaffolds, because the channels running in different directions intersect, the blood vessels diverge at the intersections. As a result, the blood vessel size and orientation degree are reduced (Figure 5b,c). Thus, even though the 1D-scaffolds have lower porosity, their intrascaffold space is advantageous for the formation of large and oriented blood vessels, which eventually leads to favorable bone formation. The findings of this study contribute to the understanding of scaffold design for the effective regeneration of bones with large and oriented blood vessels.

Scaffolds with square channels were investigated in this study. Previous studies demonstrated that channel structures, including honeycomb structure, improved oxygen diffusion and nutrient transportation and induced the tubular formation of endothelial cells aligning on the channel surface to form an endothelium [61,62,63,64]. Honeycomb structures with various geometries of unit cells, or channels, have been reported [8,9,42,43,44,45,46,47,48,65,66,67,68]. Rumpler et al., investigated the effects of scaffold channel geometry (round, square, hexagonal, and triangular channels) on tissue amplification using pre-osteoblastic cells [69]. They demonstrated that the total amounts of tissues formed in the channel were not significantly different between different channel geometries. Thus, the evaluation of osteogenesis and angiogenesis using the 1D-, 2D-, and 3D-scaffolds with square channels in this study may correlate to studies using scaffolds with channels of other geometries.

Jiang et al. compared the bone formation of radially and axially porous chitosan and HAp composite cylinders in the metaphysis of rabbit femur [70]. The channel direction of the axially porous cylinder is the same as that of the 1D-scaffold in this study. The channels of the radially porous cylinder were oriented in the direction that rotates 90° from the channels of the 1D-scaffold in this study. The axially porous cylinder was reported to be more favorable than the radially porous cylinder because the radially porous cylinder prevented the invasion of fibrous tissues and non-osteogenic cells into the channels, whereas the axially porous cylinder did not prevent the invasion. However, we have previously confirmed that our 1D-scaffold can prevent the invasion of fibrous tissues despite implantation on the calvaria, i.e., outside the bone [47,48]. In the case of intraosseous implantation (implantation in the epiphysis) in this study, the 1D-scaffolds prevented the invasion of fibrous tissues. The difference between our results and those of Jiang et al. may have resulted from differences in the osteoconductivity of the scaffolds. Jiang et al. used a composite of HAp and chitosan, which is not osteoconductive, and thus allowed fibrous tissue penetration into the axially porous cylinder. In contrast, the 1D-scaffold in this study was composed solely of carbonate apatite and had micro- and nanopores that enhance osteoconductivity [42,44], making it extremely osteoconductive and able to guide bone formation into the scaffold faster than fibrous tissues. Therefore, in this study, only bone and blood vessel formation in the 1D-scaffold were considered, because the influence of fibrous tissue invasion could be ignored. In addition, considering the fact that blood vessels in trabecular bone are abundantly present, the axial direction (the same direction as the channel of the 1D-scaffold in this study) may be more favorable than the radial direction (a 90° rotation of the channel direction of the 1D-scaffold in this study) for guiding vascular formation from the trabecular bone.

The effects of the size, architecture, geometry, and curvature of the pores or channels on bone formation have been reported [13,71,72,73,74,75,76]. However, considering the differences in cell migration, the effects of intrascaffold space have rarely been studied. The novelty of the present study is that it showed that the characteristics of intrascaffold space are important for the cell migration dimensionality and formation of large and oriented blood vessels, which eventually lead to favorable bone regeneration. In the future, the additional design of intrascaffold space dimensionalities to the structural parameters may provide more effective bone regeneration. 

Although this study revealed four-week outcomes of blood vessel and bone formation, the observation period for animal experiments is still short. To resolve this limitation, long-term studies are required in the future. Additionally, based on sample size calculations, we used four samples per group. For the analyses of blood vessel size, orientation angle, and anisotropy, the estimates of the experimental and control means and standard deviations were coincident with the experimental values. Therefore, this sample size may be considered statistically reasonable for these analyses. However, the sample size for the analysis of the amount of bone was less than that required, as the estimates of the experimental and control means were smaller than the experimental values. Furthermore, histological analysis is essential, as an analysis of blood vessel size and orientation is not possible with micro-CT data. However, micro-CT is useful in providing insight into the amount of bone; therefore, a micro-CT analysis may enhance the validity of the results of this study.

## 5. Conclusions

The 1D-scaffolds with intrascaffold spaces allowing 1D cell migration were able to induce larger and more oriented blood vessels than the 2D- and 3D-scaffolds with intrascaffold spaces allowing 2D and 3D cell migration. This may be attributed to the prevention of vessel divarication by 1D space, which cannot be achieved by 2D and 3D spaces. Furthermore, the 1D-scaffolds produced a higher percentage of new bone formation than the other two types of scaffolds, owing to their more favorable vascularization ability. This study can inspire the design and development of scaffolds for bone regeneration. In future studies, the optimization of the channel design and in vivo long-term observation of the formation of bones and blood vessels are required.

## Data Availability

The datasets that support the findings in this study are available from the corresponding author upon reasonable request.

## Supplementary Materials

The following supporting information can be downloaded at: https://www.mdpi.com/article/10.3390/ma16247518/s1, Figure S1: (a) XRD patterns of 1D-, 2D-, and 3D-scaffolds. The XRD patterns of commercial carbonate apatite and CaCO_3_ (calcite) are also shown as references. (b) FTIR spectra of 1D-, 2D-, and 3D-scaffolds and commercial hydroxyapatite.
