# Supplementary material for: Effects of Space Dimensionality within Scaffold for Bone Regeneration with Large and Oriented Blood Vessels
Source: Materials (Basel). 2023 Dec 5;16(24):7518. doi: 10.3390/ma16247518 (PMC10744811; doi:10.3390/ma16247518)
Supplement: Supplementary file 1 [file materials-16-07518-s001.zip › materials-2736373-supplementary.pdf]

Supporting Information

Effects of Space Dimension within Scaffold for Bone Regeneration with Large and Oriented Blood Vessels

Koichiro Hayashi,* Ryo Kishida, Akira Tsuchiya, and Kunio Ishikawa

Department of Biomaterials, Faculty of Dental Science, Kyushu University 3-1-1 Maidashi, Higashi-ku, Fukuoka 812-8582, Japan

*Corresponding author: Koichiro Hayashi
E-mail: khayashi@dent.kyushu-u.ac.jp

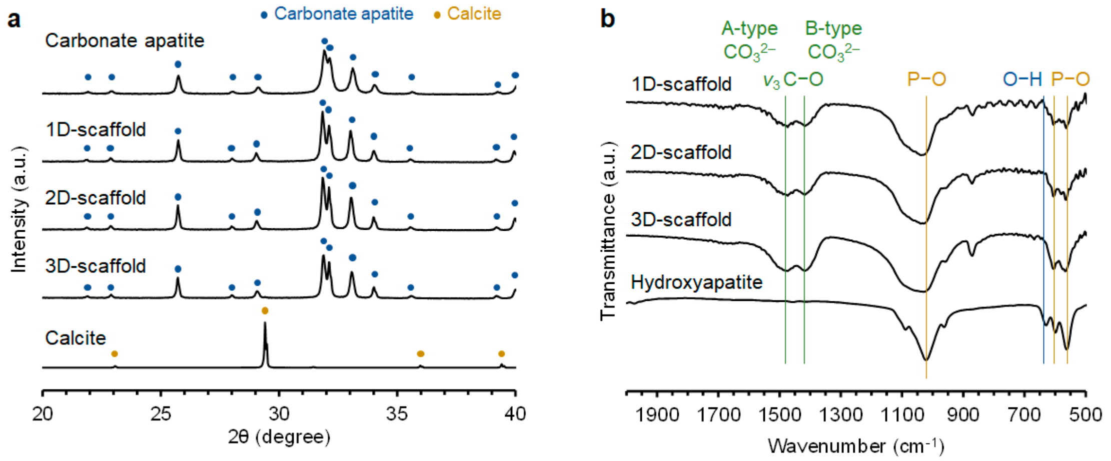

Figure S1. (a) XRD patterns of 1D-, 2D-, and 3D-scaffolds. The XRD patterns of commercial carbonate apatite and $CaCO_3$ (calcite) are also shown as references. (b) FTIR spectra of 1D-, 2D-, and 3D-scaffolds and commercial hydroxyapatite.
